# Spontaneous Emergence of Cefiderocol Resistance in *Klebsiella pneumoniae* KPC-163: Genomic and Transcriptomic Insights

**DOI:** 10.3390/antibiotics14080832

**Published:** 2025-08-15

**Authors:** Irene Luu, Vyanka Mezcord, Jenny Escalante, German M. Traglia, Marisel R. Tuttobene, Cecilia Rodriguez, Chun Fu Cheng, Quentin Valle, Rajnikant Sharma, Marcelo E. Tolmasky, Robert A. Bonomo, Gauri Rao, Fernando Pasteran, Maria Soledad Ramirez

**Affiliations:** 1 Center for Applied Biotechnology Studies, Department of Biological Science, College of Natural Sciences and Mathematics, California State University Fullerton, 800 N State College Blvd, Fullerton, CA 92831, USA; ireneluu@csu.fullerton.edu (I.L.); vmezcord@fullerton.edu (V.M.); jenni1@csu.fullerton.edu (J.E.); tuttobene@ibr-conicet.gov.ar (M.R.T.); mceci_r@hotmail.com (C.R.); mtolmasky@fullerton.edu (M.E.T.); 2 Unidad de Genómica y Bioinformática, Departamento de Ciencias Biológicas, CENUR Litoral Norte, Universidad de la República, Salto 50000, Uruguay; gtraglia@litoralnorte.udelar.edu.uy; 3 Instituto de Biología Molecular y Celular de Rosario (IBR, CONICET-UNR), Rosario 2000, Argentina; 4 Área Biología Molecular, Facultad de Ciencias Bioquímicas y Farmacéuticas, Universidad Nacional de Rosario, Rosario 2000, Argentina; 5 Centro de Referencia para Lactobacilos (CERELA), Consejo Nacional de Investigaciones Científicas y Técnicas (CONICET), Tucuman 4000, Argentina; 6 Department of Clinical Pharmacy, Alfred E. Mann School of Pharmacy and Pharmaceutical Sciences, University of Southern California, Los Angeles, CA 90089, USA; chunfuch@usc.edu (C.F.C.); qvalle@usc.edu (Q.V.); rajnikan@usc.edu (R.S.); gaurirao@usc.edu (G.R.); 7 Research Service and Geriatric Research, Education, and Clinical Center (GRECC), Louis Stokes Cleveland Department of Veterans Affairs Medical Center, Cleveland, OH 44105, USA; robert.bonomo@va.gov; 8 Departments of Medicine, Pharmacology, Molecular Biology and Microbiology, Biochemistry, Proteomics and Bioinformatics, Case Western Reserve University School of Medicine, Cleveland, OH 44106, USA; 9 CWRU-Cleveland VAMC Center for Antimicrobial Resistance and Epidemiology (Case VA CARES), Cleveland, OH 44106, USA; 10 Laboratorio Nacional/Regional de Referencia en Antimicrobianos, Instituto Nacional de Enfermedades Infecciosas, ANLIS Dr. Carlos G. Malbrán, Buenos Aires 1281, Argentina; fpasteran@gmail.com

**Keywords:** KPC, cefiderocol, *Klebsiella*, carbapenem-resistant

## Abstract

**Background/Objectives:** Carbapenem-resistant *Klebsiella pneumoniae* (CRKP) is an urgent public health threat due to its rapid dissemination and resistance to last-line antibiotics. Cefiderocol (FDC), a novel siderophore cephalosporin, targets resistant Gram-negative pathogens by exploiting bacterial iron uptake mechanisms. However, resistance to FDC is emerging among *Klebsiella pneumoniae* carbapenemase (KPC)-producing *K. pneumoniae* strains. This study characterizes a spontaneous FDC-resistant subpopulation (IHC216) derived from a KPC-producing strain (KPNMA216) using comprehensive genomic, transcriptional, and phenotypic analyses. **Methods**: Given the whole-genome sequencing results, where mutations were identified in genes involved in transcriptional regulation and membrane permeability (*ompC*) among others, in the present work we further explore their potential implications and conduct a more detailed analysis of the IHC216 genome. A qRT-PCR analysis highlighted significant downregulation of classical siderophore-mediated iron acquisition systems (*fepA*, *cirA*, *iroN*) and upregulation of alternative iron uptake pathways (*iucA*, *fiU*), reflecting a switch in iron acquisition strategies. **Results**: A notable downregulation of *bla*_KPC-163_ correlated with restored susceptibility to carbapenems, indicating collateral susceptibility. Altered expressions of *pbp2* and *pbp3* implicated adaptive changes in cell wall synthesis, potentially affecting FDC resistance mechanisms. Furthermore, enhanced oxidative stress responses via upregulated *sodC* expression and increased capsule production were observed. **Conclusions**: These findings underscore the complex interplay of genetic and transcriptional adaptations underlying FDC resistance, highlighting potential therapeutic vulnerabilities.

## 1. Introduction

Carbapenem-resistant *K. pneumoniae* (CRKP) has emerged as a significant global health concern due to its high level of resistance to multiple antibiotics, including carbapenems, which are often considered last resort treatments for severe infections. The rapid dissemination of these resistant strains poses substantial challenges in clinical settings, particularly in intensive care units (ICUs), where outbreaks can increase morbidity and mortality among critically ill patients [1]. Treatment options for infections caused by CRKP are increasingly limited, particularly as these strains develop resistance to last-line antibiotics such as ceftazidime/avibactam and colistin [2]. Studies have shown that combination therapy may be more effective than monotherapy in treating CRKP infections, with significantly more treatment failures observed in cases that received monotherapy compared to those that received combination therapy [3].

Cefiderocol (FDC) is a novel siderophore cephalosporin designed to combat infections caused by carbapenem-resistant Gram-negative bacteria, including CRKP strains. Its unique mechanism involves utilizing the bacterial iron transport system to enter the cell, thereby inhibiting cell wall synthesis [4,5]. However, emerging evidence indicates that apart from mutations in iron uptake systems, certain New Delhi metallo–β–lactamase (NDM) and *Klebsiella pneumoniae* carbapenemase (KPC)-producing *K. pneumoniae* strains are developing resistance to FDC [5]. Regarding KPC, specific variants of KPC have been linked with FDC resistance [6,7,8]. Hobson et al. revealed that KPC-31 confers cross-resistance to both ceftazidime-avibactam and FDC [9]. Additionally, a significant inoculum effect on FDC was observed, indicating that higher bacterial loads may diminish the antibiotic’s efficacy [9].

Our group has recently found collateral-resistance to FDC and cefepime/zidebactam (FPZ) in eight and three of the *Klebsiella* isolates carrying different KPC variants, respectively [10]. We observed that five strains exhibited the occurrence of colonies within the inhibition ellipse zones of FDC strips, suggesting the occurrence of heteroresistance or spontaneous resistant mutants. This phenomenon has been observed in Gram-negative organisms and has been linked to FDC resistance and to last resort antibiotics such as colistin, meropenem, and ceftazidime-avibactam (CZA) [11,12,13,14].

To our knowledge, the role of *dksA* mutations in cefiderocol resistance has not been described previously in KPC-producing *K. pneumoniae*. Therefore, we aimed to test the hypothesis that *dksA* mutations, in combination with transcriptional changes in iron acquisition pathways, may contribute to metabolic reprogramming associated with cefiderocol resistance. To address this, we characterize a spontaneously emerged FDC-resistant subpopulation (IHC216) by conducting a comprehensive genomic, transcriptional, and phenotypic analysis in comparison to its parental strain.

## 2. Results

### 2.1. Whole Genome Sequencing Analysis

As previously reported by Hamza et al. [10], the comparison of KPNMA216 and IHC216 demonstrated that there are no gene content differences; however, we found 14 mutations, including 5 SNPs and 9 InDels. Remarkably, we have observed a deletion at the 456-nucleotide position in the *dksA* gene, a global transcription regulator, that it was reported to make a significant phenotype global transcription regulator, and that it was reported to make significant phenotype changes in different bacterial species. There are a few differences, with the main differences at the SNP/InDel level.

To further explore the distinct features of the KPNMA216, genomic comparative analysis was performed. A detailed comparative analysis of the KPNMA216 genome (wild-type strain) with all 477 *K. pneumoniae* ST14 genomes available in the Genome NCBI database (Table S1) revealed 1644 conserved gene families and 4 unique genes in KPNMA216. Among the unique genes, we found the insertion sequence IS*Apu2* and the fragment of the transposase of IS*Kpn54*, *traD* (conjugative system), and *tssK* (Type VI Secretion System) genes.

**Figure 1:**
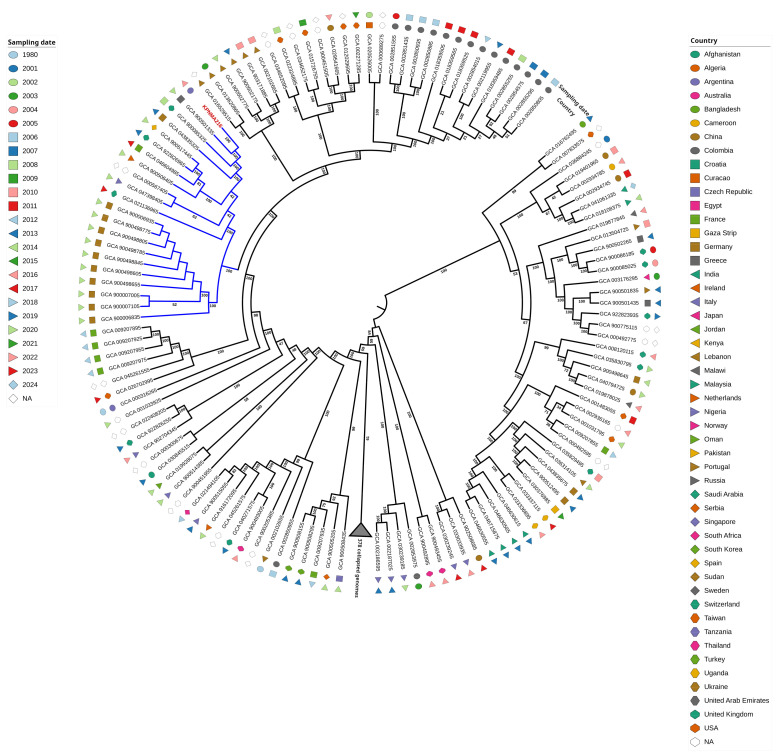
A core-genome phylogenetic analysis was performed using the KPNMA216 genome and 477 *Klebsiella pneumoniae* ST14 genomes available in the NCBI Genome database. The KPNMA216 strain clustered within a monophyletic group located in a clade that included isolates recovered from various regions, including Germany, Greece, Turkey, France, the United Kingdom, and Italy.

A core-genome phylogenetic analysis was performed of the KPNMA216 genome and 477 *K. pneumoniae* ST14 genomes available in Genome NCBI Database (Figure 1 and Figure S1). From the phylogenetic analysis of *K. pneumoniae*, the KPNMA216 strain was clustered in a monophyletic group that included isolates recovered from Europe (Germany, Greece, Turkey, France, Italy, and the United Kingdom) (Figure 1). A temporal trend was observed within the KPNMA216 clade, with the most isolates being recovered in 2014. KPNMA216 was isolated in 2022, potentially indicating a recent emergence and suggesting that the strain may have originated in Europe (Figure 1).

Fifty-two genes associated with virulence phenotype were identified using the VFDB database. Among the virulence genes in *K. pneumoniae*, we found genes involved in the type VI secretion system, fimbrial biogenesis, etc. [15,16,17]. The capsular polysaccharide is a crucial virulence factor in Gram-negative bacteria, enabling resistance to the bactericidal activity of the complement system. *K. pneumoniae* KPNMA216 contains the capsular polysaccharide biosynthesis loci (KL, K locus) and LPS loci (OCL, OC locus). Comparative analysis of the KL structure in KPNMA216 showed a 99% nucleotide identity and 100% coverage with the KL2 type. The OCL locus, responsible for O antigen synthesis, was identified as O1/02v2 (Table S2 with Virulence genes)

### 2.2. Collateral Susceptibility to Carbapenems in the FDC-Resistant Mutant

When the minimum inhibitory concentration (MIC) for cefiderocol (FDC) in IHC216 was evaluated, it increased from 8 mg/L in KPNMA216 to 32 mg/L (Table S3, Figure S2), confirming the development of FDC resistance. This finding indicates the emergence of stable FDC-resistant colonies within the inhibition ellipse (intracolonies) in KPC-producing strains.

To investigate potential collateral susceptibility and collateral resistance, MIC values for KPNMA216 and IHC216 were compared across a panel of antimicrobial agents. IHC216 exhibited a notable decrease in resistance to carbapenems, with meropenem MIC decreasing from 32 mg/L to 0.5 mg/L and imipenem MIC decreasing from 48 mg/L to 3 mg/L (Table 1). A similar trend was observed for imipenem/relebactam (from 2 mg/L to 0.75 mg/L) and meropenem/varbobactam (from 2 mg/L to 0.064 mg/L), indicating restored susceptibility to these β–lactam/β–lactamase inhibitor combinations (Table 1).

However, susceptibility to cefepime/zidebactam (FPZ) remained unchanged (MIC = 16 mg/L). Ciprofloxacin MIC decreased slightly (0.094 mg/L to 0.064 mg/L), but no significant changes were observed for ceftolozane/tazobactam, aztreonam, amikacin, or colistin. Interestingly, tigecycline MIC increased from 0.25 mg/L to a range of 0.75 to 1 mg/L, suggesting a potential fitness cost associated with the observed carbapenem susceptibility restoration (Table 1).

These findings highlight differential effects of the resistance mechanism on susceptibility patterns across various antimicrobial classes and underscore the complex interplay between β–lactam resistance and collateral susceptibility in KPC-producing *K. pneumoniae*.

### 2.3. Molecular and Transcriptional Adaptations Underlying FDC Resistance in the IHC216 Mutant

**Figure 2:**
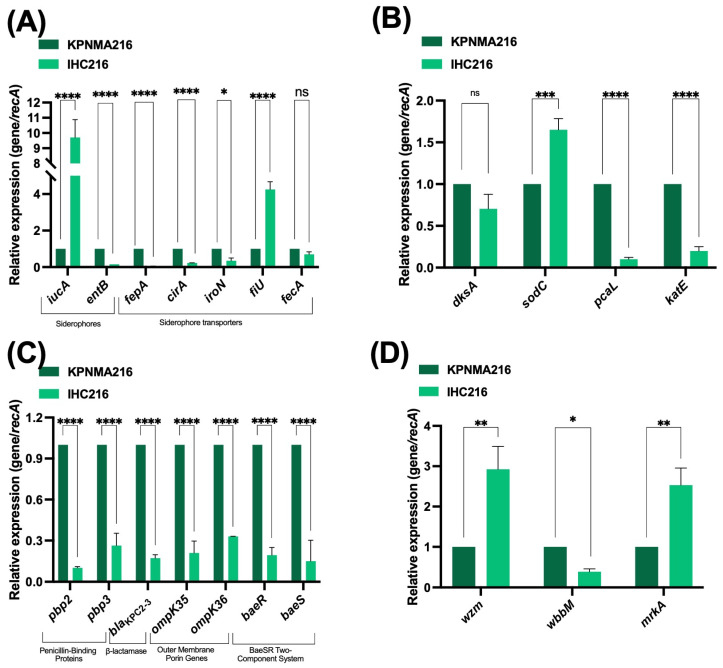
Expression of genes coding for siderophores (*iucA* and *entB*) and siderophores transporters (*fepA*, *cirA*, *iroN*, *fiU* and *fecA*) (**A**), *dksA* and genes regulated by DksA (*sodC*, *pcaL* and *katE*) (**B**), *pbp2* and *pbp3*, β-lactamase *bla*_KPC2–3_, outer membrane porins *ompK35* and *ompK36*, and the BaeRS two-component system (**C**), *wzm*, *wbbM*; and *mrkA* (**D**) in the KPNMA216 and IHC216 strains. The data shown for qRT-PCR are mean ± SD. Fold changes were calculated using ΔΔCt analysis. At least three independent biological samples were tested using four technical replicates. Statistical significance (*p* < 0.05) was determined by two-way ANOVA followed by Tukey’s multiple comparison test using GraphPad Prism Version 10.5.0 (GraphPad software, San Diego, CA, USA). Significance was indicated by: * *p* < 0.05, ** *p* < 0.01, *** *p* < 0.001, and **** *p* < 0.0001.

To further investigate the factors contributing to FDC resistance in the emergent resistant colony, we analyzed the preliminary whole-genome comparative sequence data of the IHC216 strain against the wild-type [10]. The analysis identified 14 non-synonymous mutations ([10] and Table S3), of which seven directly affected coding regions. Among these, several mutations occurred in genes with known functions, including *dksA*, *ompC*, *licC_7*, *gmuC*, and *lacE*. These mutations suggest potential alterations in transcriptional regulation, membrane permeability, and carbohydrate transport, which may contribute to the observed resistance phenotype. Notably, no mutations were detected in iron uptake systems or siderophore production, indicating that FDC resistance in IHC216 is not driven by mutations in those genes. To further assess the molecular mechanisms underlying resistance, we performed quantitative real-time PCR (qRT-PCR) to evaluate the expression of genes associated with iron uptake, antibiotic resistance, cell wall synthesis, oxidative stress response, and aromatic compound catabolism in IHC216 compared to the parental strain (Figure 2 and Table S4). The analysis of iron uptake-associated transcripts revealed a marked downregulation of *fepA* and *cirA*, which encode siderophore receptors [18,19,20], as well as *iroN*, an enterobactin receptor. Additionally, a significant decrease in *entB* [21] expression was observed, indicating reduced enterobactin biosynthesis. These changes suggest a reduced expression of certain iron acquisition systems or regulatory adjustments in the IHC216 strain. Conversely, there was a significant upregulation of *iucA*, which encodes a key enzyme in aerobactin synthesis [22], and *fiU*, a siderophore receptor [18], indicating a shift toward alternative iron uptake mechanisms in the IHC216 environment. In contrast, *fecA* expression remained unchanged, suggesting that the ferric citrate transport system is not significantly affected under these conditions (Figure 2A, Table S4).

The expression of *dksA*, a key regulator influencing iron homeostasis and mutated in the IHC216, was downregulated, suggesting that the mutation may be affecting the expression of this gene, leading to the observed changes in iron metabolism (Figure 2B, Table S4). Given DksA’s role in bacterial stress responses and iron acquisition, its repression may impact siderophore production and iron uptake pathways, potentially influencing FDC resistance mechanisms.

Additionally, genes involved in oxidative stress responses, known to be regulated by DksA, exhibited notable changes. Specifically, *sodC*, which encodes a copper–zinc superoxide dismutase (Cu/Zn-SOD) involved in neutralizing reactive oxygen species, was significantly upregulated. This suggests an enhanced oxidative stress defense mechanism, likely in response to increased oxidative challenges in the presence of FDC. In addition, a metabolic shift was observed in aromatic compound catabolism, pathway control by *dksA* in *A. baumannni* [23,24], as *pcaL*, which encodes a key enzyme involved in the degradation of phenolic acids, was significantly downregulated (Figure 2B, Table S4). Moreover, the expression of *katE* was found to be significantly lower in strain IHC216 compared to the parental strain. *katE* encodes the catalase-peroxidase enzyme, which plays a role in the bacterial oxidative stress response by breaking down hydrogen peroxide (H_2_O_2_) into water and oxygen. This enzyme helps protect *Klebsiella* from oxidative damage, especially under stress conditions such as exposure to reactive oxygen species (ROS) or during host immune responses [25]. These results suggest that strain IHC216 exhibits an impaired oxidative stress response, potentially affecting its ability to detoxify hydrogen peroxide (Figure 2B, Table S4).

In terms of antibiotic resistance, the expression of several critical genes was significantly reduced. *bla*_KPC-163_, encoding carbapenemase enzymes responsible for resistance to carbapenems [26], showed notable downregulation, which can explain the collateral susceptibility of IHC216 towards carbapenems (Table 1). However, the outer membrane porins coding genes, *ompK35* and *ompK36* [27], which facilitate the influx and efflux of antibiotics, were also downregulated, potentially altering membrane permeability and reducing resistance potential supporting cefepime/zidebactam (FPZ) resistance. Furthermore, the two-component regulatory system genes *baeR* and *baeS*, associated with multidrug resistance and membrane stress responses, exhibited decreased expression. These findings suggest a diminished antibiotic resistance capability in IHC216, possibly due to altered membrane dynamics or regulatory mechanisms. Regarding cell wall synthesis, the expression of *pbp2* and *pbp3*, which encode penicillin-binding proteins essential for peptidoglycan synthesis and cell wall integrity, was significantly reduced. This downregulation may reflect alterations in cell wall remodeling in the IHC216, which can affect FDC activity (Figure 2C, Table S4). This suggests a reduced utilization of aromatic compounds in the IHC216 strains, possibly reflecting broader metabolic adaptations associated with FDC resistance.

In the IHC216 strain, quantitative real-time PCR analysis revealed significant alterations in the expression of key genes associated with biofilm formation and capsule when compared to the parental strain. Specifically, the *mrkA* and *wzm* genes exhibited upregulation, while the *wbbM* gene was downregulated (Figure 2D, Table S4). The *mrkA* gene encodes the major structural subunit of type 3 fimbriae, which are crucial for biofilm formation and adherence to surfaces [28]. Wzm is involved in the transport of lipopolysaccharide (LPS) components across the inner membrane, playing a role in the assembly of the bacterial outer membrane [29]. Finally, WbbM is associated with the synthesis of D-galactan I, a component of the O-antigen in LPS [30]. These expression changes in IHC216 highlight a potential shift in its pathogenic profile, with implications for its biofilm-forming ability and interaction with host defenses.

### 2.4. Increased Capsule and Biofilm Formation Was Seen in the FDC-Resistant Mutant

A key virulence factor in *K. pneumoniae* is the capsule, which plays a critical role in immune evasion, resistance to antimicrobial peptides, and protection against phagocytosis by host immune cells [31,32]. Capsule production has been linked to increased antibiotic resistance, pathogenicity, and survival in hostile environments, including resistance to complement-mediated killing. Capsule density was evaluated, showing higher capsule production in the IHC216 strains compared to its parental strain (Figure S3A).

In addition, biofilm production, which is known to contribute to antimicrobial resistance, was performed for both the wild-type strain and the FDC-resistant mutant (IHC216). We observed that the IHC216 mutant exhibited increased biofilm formation compared to the wild-type KPNMA216 (Figure S3B).

These results highlight potential trade-offs between capsule expression and biofilm formation in response to selective pressures in the emergence of a mutant population. Further investigation into the genetic mechanisms underlying these changes will provide deeper insights into the adaptation strategies of *K. pneumoniae.*

## 3. Discussion

The development of cefiderocol (FDC) resistance in KPC-producing *K. pneumoniae* represents a multifaceted adaptation involving genetic mutations, transcriptional reprogramming, and metabolic shifts. Previous reports have shown the FDC resistance in KPC-producing *K. pneumoniae* [6,18,33]. One mechanism involves the acquisition of plasmids carrying the ferric citrate transport (FEC) system. The presence of two co-resident plasmids—pKpQIL, which harbors variants of the *bla*_KPC_ carbapenemase gene, and pKPN, containing the FEC system—can decrease FDC susceptibility in *K. pneumoniae* clinical isolates [18]. Mutations in the CirA receptor have also been reported in FDC-resistant hypervirulent *K. pneumoniae* [19]. Additionally, co-production of carbapenemases such as NDM and KPC can further increase FDC minimum inhibitory concentrations (MICs) [34]. Through whole-genome sequencing and transcriptomic analyses of the FDC-resistant subpopulation (IHC216), we identified mutations in genes related to transcriptional regulation, membrane permeability, iron uptake, antibiotic resistance, and carbohydrate transport. These changes likely contribute to FDC resistance but also introduced collateral susceptibility to carbapenems, as evidenced by the significant reduction in meropenem (32 mg/L to 0.5 mg/L) and imipenem (48 mg/L to 3 mg/L) MICs.

Collateral susceptibility, defined as the unintended restoration of antibiotic sensitivity [35], was observed in IHC216, likely due to the downregulation of *bla*_KPC-163_. Reduced expression of this KPC variant suggests that mutations conferring FDC resistance impose a regulatory cost on KPC expression. A similar phenomenon has been reported in KPC-31-producing strains, where a single amino acid substitution resulted in cross-resistance to both ceftazidime–avibactam and FDC [9]. Previous studies also reported that heteroresistant subpopulations of KPC-*K. pneumoniae* may exhibit increased meropenem susceptibility, in agreement with our findings [36]. This effect could mask carbapenemase production, complicating phenotypic susceptibility testing and potentially affecting treatment decisions.

Interestingly, genes coding penicillin-binding proteins *pbp2* and *pbp3* were also downregulated, suggesting potential cell wall remodeling in response to FDC exposure. As FDC inhibits peptidoglycan biosynthesis, these changes may represent an adaptive mechanism that enhances carbapenem susceptibility. In addition, the downregulation of these genes in the IHC condition may reflect a shift in bacterial physiology toward slower growth or altered division cycles. Additionally, reduced *pbp2* and *pbp3* expression may provide protection against oxidative stress, consistent with reports in biofilm-forming bacteria, where PBPs are differentially expressed to adapt to the biofilm matrix and reduced metabolic activity [37]. Furthermore, reduced expression of *ompK35* and *ompK36*, encoding β–lactam influx porins [27], could alter drug permeability, explaining why IHC216 remained resistant to cefepime/zidebactam despite restored carbapenem susceptibility.

Another important observation in IHC216 was the differential expression of iron acquisition genes. Given that FDC is a siderophore cephalosporin, bacterial iron uptake pathways play a central role in both FDC susceptibility and resistance development. The downregulation of *fepA*, *cirA*, *iroN*, and *entB* in IHC216 suggests reduced relevance of catecholate siderophore receptors, potentially due to an environmental shift reducing iron competition. This finding is consistent with studies showing that bacteria downregulate siderophore receptor expression in iron-rich conditions or when alternative iron uptake systems are activated [38,39]. Conversely, upregulation of *iucA* and *fiU* indicates a shift toward aerobactin-mediated iron acquisition, consistent with reports demonstrating that aerobactin expression is favored under oxidative stress [40]. Additionally, the mutation and low expression of *dksA* in IHC216 suggest that this global regulator plays a central role in modulating iron metabolism. Previous work in *Salmonella enterica* has shown that DksA regulates iron homeostasis and oxidative stress responses, with its deletion leading to dysregulated iron uptake and increased sensitivity to reactive nitrogen species [41]. Furthermore, this effect extends to other metabolic pathways, as evidenced by the downregulation of *pcaL*, involved in aromatic compound metabolism, suggesting broader metabolic reprogramming.

We have also observed a significant upregulation of *sodC*, which encodes a copper-zinc superoxide dismutase (Cu/Zn-SOD), a major antioxidant defense enzyme in the IHC216. Increased oxidative stress resistance in IHC216 suggests that FDC exposure induces a reactive oxygen species (ROS)-rich environment, requiring an enhanced detoxification response. This agrees with previous findings that dense bacterial populations experience heightened oxidative stress due to metabolic activity and host immune responses [42,43].

In addition to oxidative stress defenses, capsule production was significantly increased in IHC216. The capsule plays a key role in immune evasion and antimicrobial resistance [31,32], and its upregulation in IHC216 may serve as a compensatory adaptation to balance FDC-induced permeability and iron acquisition shifts. Notably, capsule production has been previously associated with increased FDC resistance, suggesting that enhanced polysaccharide biosynthesis might limit drug penetration into the bacterial cell.

Lastly, biofilm formation was also significantly increased in IHC216. While biofilms contribute to antimicrobial resistance, their development is often influenced by capsule production, with a potential trade-off between biofilm adherence and immune evasion. Recent studies suggest that capsule-deficient strains tend to form more robust biofilms, while hypermucoid variants rely more on capsular protection for survival.

This study highlights the complex regulatory and metabolic adaptations underlying FDC resistance in KPC-producing *K. pneumoniae*. This work showed that apart from recognized resistance mechanisms, IHC216 displayed collateral susceptibility to carbapenems, which could inform novel treatment strategies leveraging this vulnerability. The downregulation of *bla*_KPC-163_, alterations in *pbp* expression, and outer membrane modifications suggest that targeting cell wall synthesis pathways in combination with carbapenems may restore treatment efficacy. Moreover, the observed iron uptake reprogramming in IHC216 highlights potential therapeutic avenues focusing on siderophore-mediated drug delivery. Given that aerobactin synthesis was upregulated, targeting this pathway with siderophore-conjugated antimicrobials could offer a more effective strategy against FDC-resistant strains. Additionally, the induction of oxidative stress defense pathways suggests that combining FDC with ROS-enhancing agents could further sensitize resistant populations.

Recent studies have also reported heteroresistance to FDC in *K. pneumoniae* and *Acinetobacter baumannii*, where subpopulations exhibit transient resistance under host-associated conditions. Our laboratory previously demonstrated that human pleural fluid could induce cefiderocol heteroresistance in carbapenem-resistant *A. baumannii*, further supporting the notion that host environments drive dynamic resistance evolution. Similarly, mutations in *cirA*—a FDC receptor—were identified during in vivo evolution of a *K. pneumoniae* ST512 strain, leading to high-level FDC resistance.

In summary, this study provides novel insights into the genetic, transcriptional, and phenotypic landscape of FDC resistance in KPC-producing *K. pneumoniae*. The interplay between iron homeostasis, oxidative stress defense, and membrane permeability highlights multiple vulnerabilities that could be therapeutically exploited. The collateral susceptibility to carbapenems supports the design of optimized combination therapies, which may help circumvent FDC resistance by exploiting this trade-off. Further research should aim to experimentally confirm the causal link between the identified mutations and FDC resistance, for example, through targeted mutagenesis or complementation assays. Future investigations should address the clinical implications of these resistance mechanisms to guide treatment strategies against multidrug-resistant *K. pneumoniae* and cautiously decide the optimal treatment.

## 4. Materials and Methods

### 4.1. Bacterial Strains

The KPC-producing *K. pneumoniae* KPNMA216 and the KPNMA216 FDC-resistant subpopulation (IHC216) strains were further analyzed in the present study (Table S3) [10]. The IHC216 strain was recovered within the inhibition ellipse zones of FDC strips during the MIC determination of the KPNMA216 parental strain (Figure S2). Copies of the IHC isolates were kept at −80 °C as Luria–Bertani (LB) broth containing 20% glycerol stocks that were plated on Cystine–Lactose–Electrolyte-Deficient (CLED) medium (Beckton Dickinson, Franklin Lakes, NJ, USA) and used within 24 h after overnight (16–18 h) incubation at 37 °C. The resistance phenotype stability was determined after 10 daily subcultures in CLED antibiotic-free plates.

### 4.2. Whole Genome Sequencing

The whole-genome sequencing data of the parental strain KPNMA216 and the IHC216 generated in our previous study [10] were further used for detailed resistome analysis. The fastq files, assemblies, and annotations are deposited in Zenodo (https://zenodo.org/records/14019667, accessed on 6 August 2025). The quality, sequence, and assembly were found using FASTQC and QUAST software version 0.12.1. Stat data of genome sequencing and assembling (fastqc file and quast table) are deposited in another Zenodo repository (https://zenodo.org/records/16728904, accessed on 30 July 2025). The 16S rDNA sequences comparison of KPNMA216 and the IHC216 confirms 100% identity (Figure S4). In addition, the Average Nucleotide Identity (ANI), calculated using the JSpeciesWS tool version 5.0.2 [44], showed 99.9% identity to *K. pneumoniae*.

The genomes were annotated using PROKKA (version 1.14.5) [45]. The ortholog functional assignment was performed using EggNOG v2.0 (default parameter) [46]. The pangenome analysis was performed using Roary software version 3.13.0 [47]. To assess core genome phylogeny, we used 477 *K. pneumoniae* ST14 sequences from a total of 23,578 *K. pneumoniae* genomes available in the GenBank (Table S1). Core genome phylogeny analysis was performed using the maximum likelihood method, implemented with IQtree2 using default parameters [41]. Bootstrap method was performed for phylogenetic validation using IQtree2 Software version 2.4.0.

tRNA and ncRNA predictions were conducted using tRNAscan-SE (version 1.3) and Infernal (version 1.1.5) software, respectively [43], and the Multilocus Sequence Typing (MLST) profile, OC and K locus, ICEKp-associated virulence loci, colibactin (*clb*), salmochelin (*iro*), and hypermucoidy (*rmpA*) were determined using Kleborate software version 3.2.4 [48]. Other virulence genes were predicted using the VFDB database.

### 4.3. RNA Extraction and Transcriptional Analysis Using Quantitative RT-qPCR

Overnight cultures of KPNMA216, and IHC216 were diluted 1:10 in LB and incubated with agitation for 18 h at 37 °C. RNA was extracted from each sample using the Direct-zol RNA Kit (Zymo Research, Irvine, CA, USA) following the manufacturer’s instructions. Total RNA extractions were performed using three independent biological replicates for each condition.

The RNA samples obtained were subjected to DNase treatment (Thermo Fisher Scientific, Waltham, MA, USA) following the manufacturer’s instructions; afterwards, a PCR amplification of the 16S rDNA gene was performed to confirm there was no DNA contamination.

In addition, the extracted DNase-treated RNA was used to synthesize cDNA according to the iScript™ Reverse Transcription Supermix for qPCR reagents (Bio-Rad, Hercules, CA, USA) manufacturer’s protocol. The cDNA concentrations were adjusted to 50 ng/μL, and qPCR was conducted using the qPCRBIO SyGreen Blue Mix Lo-ROX following the manufacturer’s protocol (PCR Biosystems, Wayne, PA, USA).

Transcriptional analysis of KPNMA216 and IHC216 was performed using specific primers (Table S5). At least three independent cDNA replicates were tested in triplicate using the CFX96 Touch™ Real-Time PCR Detection System (Bio-Rad, Hercules, CA, USA). Transcriptional levels of each sample were normalized to the transcriptional level of *recA*. The relative quantification of gene expression was performed using the comparative threshold method 2^−ΔΔCt^ [49]. Differences were determined by ANOVA followed by Tukey’s multiple comparison test (*p* < 0.05) using GraphPad Prism (GraphPad Software, San Diego, CA, USA).

### 4.4. Antimicrobial Susceptibility Testing

The minimum inhibitory concentrations (MICs) for meropenem (MEM), imipenem (IMP), imipenem/relebactam (I/R), meropenem/vaborbactam (M/V), cefepime/zidebactam (FPZ), ciprofloxacin (CIP), ceftolozane/tazobactam (C/T), aztreonam (ATM), amikacin (AK), tigecycline (TGC), and colistin (CS) were determined using commercial E-strips (Liofilchem S.r.l., Roseto degli Abruzzi, Italy). The MICs for colistin were determined using the broth microdilution method. All procedures were carried out in accordance with the manufacturer’s instructions and met the standards of the Clinical and Laboratory Standards Institute (CLSI) [50] and the European Committee on Antimicrobial Susceptibility Testing (EUCAST) (https://www.eucast.org/clinical_breakpoints, accessed on 13 August 2025). The CLSI breakpoint for cefepime was used for FPZ categorization. Quality control strains, such as *Escherichia coli* ATCC 25922 and the *K. pneumoniae* ATCC strains, were included in the experiments. Each strain was tested at least in duplicates.

### 4.5. Capsule and Biofilm

Capsule production assay was performed according to Valcek et al. [51]. Briefly, overnight bacterial cultures (1 mL) of KPNMA216 and IHC216 were transferred to 1.5 mL microtubes and centrifuged at 7000× *g* for 2 min. After removing the supernatant, the pellet was resuspended in 1 mL of phosphate-buffered saline (PBS). A total of 875 µL of the PBS-resuspended bacteria was then mixed with 125 µL of Ludox LS colloidal silica (30% [wt/wt] suspension in H_2_O; Merck). The mixture was centrifuged at 12,000× *g* for 30 min, and the resulting band position was immediately photographed. The distance from the center of the band to the bottom of the microtube was measured. Each experiment was performed in triplicate, and results were statistically analyzed by calculating the standard deviation from the mean of biological replicates.

Biofilm assays were performed as previously described [52,53]. KPNMA216 and IHC216 cells were cultured in LB broth and incubated in the tubes at 37 °C for 24–48 h without shaking (static). Experiments were performed in triplicate, with at least three technical replicates per biological replicate. Statistical analysis was performed using the test with GraphPad Prism, and a *p*-value <0.05 was considered statistically significant.

## Figures and Tables

**Table 1:** The minimum inhibitory concentration (MIC) for KPNMA216 and IHC216. The MIC was performed following manufacturer’s recommendations (Liofilchem S.r.l., Roseto degli Abruzzi, Italy).

MIC (mg/L)	KPNMA 216	IHC216
Meropenem (MEM)	32	0.50
Imipenem (IMP)	48	3
Imipenem/Relebactam (I/R)	2	0.75
Meropenem/Vaborbactam (M/V)	2	0.064
Cefepime/Zidebactam (FPZ)	16	16
Ciprofloxacin (CIP)	0.094	0.064
Ceftolozane/Tazobactam (C/T)	>256	>256
Aztreonam (ATM)	24	24
Amikacin (AK)	1.5	1.5
Tigecycline (TGC)	0.25	0.75–1
Colistin (CS)	1.5	1.5

## Data Availability

The original contributions presented in this study are included in the article/Supplementary Materials. Further inquiries can be directed to the corresponding author.

## Supplementary Materials

The following supporting information can be downloaded at: https://www.mdpi.com/article/10.3390/antibiotics14080832/s1. Supplementary Materials associated with this article can be found, in the online version. Figure S1. Core-genome phylogenetic analysis of the KPNMA216 genome in comparison with 477 publicly available *Klebsiella pneumoniae* ST14 genomes retrieved from the NCBI Genome database. Figure S2. The IHC216 strain was recovered from within the inhibition ellipse zones of FDC strips during the MIC determination of the parental KPNMA216 strain. The IHC216 colony is shown marked with a red circle. Figure S3. (A) Capsule density in KPNMA216 and IHC216 strains. (B) Biofilm formation in tubes quantified by crystal violet in both strains. Statistical analysis was determined by *t* test (*p* < 0.05), using GraphPad Prism (GraphPad software, San Diego, CA, USA); (*) indicates *p* < 0.05. Figure S4. 16S rRNA gene sequence alignment of *K. pneumoniae* strains KPNMA216 and IHC216. Table S1 Collection of 477 publicly available *Klebsiella pneumoniae* ST14 genomes retrieved from the NCBI Genome database. Table S2. Virulence genes prediction in *K. pneumoniae* using the Blastp method. Table S3. Parental KPNMA216 and IHC216 relevant mutations associated with antibiotic resistance and MIC. Table S4. Level of expression of different genes obtained by qRT-PCR in KPNMA216 and IHC216. Table S5. qRT-PCR primers used in this study.
