# Supplementary material for: Spontaneous Emergence of Cefiderocol Resistance in Klebsiella pneumoniae KPC-163: Genomic and Transcriptomic Insights
Source: Antibiotics (Basel). 2025 Aug 15;14(8):832. doi: 10.3390/antibiotics14080832 (PMC12382882; doi:10.3390/antibiotics14080832)
Supplement: Supplementary file 1 [file antibiotics-14-00832-s001.zip › Table S3.pdf]

Table S3 Parental KPNMA216 and IHC216 relevant mutations associated with antibiotic resistance and MIC

Strain ID	KPC variant	Relevant mutations detected	Minimum Inhibitory Concentration (mg/L)		
			FDC	CZA	FPZ
KPNMA216	KPC-163	Wild-type strain	8	24	16
IHC216	KPC-163	*ompC:* 311K (TAG → AAG), *dksA*: deletion (55/456 nt)	32	32	16

FDC, cefiderocol; CZA, ceftazidime avibactam; FPZ: cefpime zidebactam. Red cells correspond to MIC values classified as resistant according to CLSI criteria
