# Supplementary material for: Spontaneous Emergence of Cefiderocol Resistance in Klebsiella pneumoniae KPC-163: Genomic and Transcriptomic Insights
Source: Antibiotics (Basel). 2025 Aug 15;14(8):832. doi: 10.3390/antibiotics14080832 (PMC12382882; doi:10.3390/antibiotics14080832)
Supplement: Supplementary file 1 [file antibiotics-14-00832-s001.zip › Table S4 log 2FC 19.02.25 KPNMA216&IHC qPCR.pdf]

Table S4. Level of expression of different genes obtained by qRT-PCR in KPNMA216 and IHC216

Gene name	KPNMA216 IHC (Log_2FC)
irp1	-1.16
iucA	3.28
entB	-2.68
fepA	-4.43
cirA	-2.13
iroN	-1.52
fiU	1.99
fecA	-0.50
dksA	-0.69
sodC	0.72
pcaL	-3.31
katE	-2.33
pbp2	-3.32
pbp3	-1.93
bla_{KPC2-3}	-2.29
ompK35	-2.26
ompK36	-1.20
baeR	-2.10
baeS	-2.74
wzm	1.55
wbbM	-1.37
mrkA	1.33

$Log_2FC > 1$ ($p<0.05$) $Log_2FC < -1$ ($p<0.05$)

Log_2FC (0-1) or p>0,05 Log_2FC (-1-0) or p>0,05
