# Supplementary material for: Spontaneous Emergence of Cefiderocol Resistance in Klebsiella pneumoniae KPC-163: Genomic and Transcriptomic Insights
Source: Antibiotics (Basel). 2025 Aug 15;14(8):832. doi: 10.3390/antibiotics14080832 (PMC12382882; doi:10.3390/antibiotics14080832)
Supplement: Supplementary file 1 [file antibiotics-14-00832-s001.zip › Table S5.pdf]

Table S5. qRT-PCR primers used in this study.

Primers	Sequence (5'–3')
fepA-Fw	CGACGTCTCGGAGATCATT
fepA-Rv	GATATCAATCTGGCGGTTGTT
cirA-Fw	CGACGGCAAGTATGTCCTG
cirA-Rv	GCTCAGGTTGACCGGATCT
iucA-Fw	TTTCCTGCTCATCTGGTCAC
iucA-Rv	CTGGCAGAAAAAGTTGATGC
fiU-Fw	AGTATGCCGCACTCAACTCC
fiU-Rv	GGGTACCGCTGTCGGTAGTA
dksA-Fw	TCAGCCTGGAACTGCGTAAC
dksA-Rv	GGATTCGCAGTAGCCGAAGT
sodC-Fw	TTACGATCCGCAGCATACCG
sodC-Rv	CAATATTCGGCTGGTCGGCT
pcaL-Fw	CCATCGTAGAGGTCTCGCAG
pcaL-Rv	GTTTTCCAGCGAGAAGACGC
pbp2-Fw	GTCAGACCGGCTACGAAGAG
pbp2-Rv	TGAGGTCGAGGGTGAGGTAG
pbp3-Fw	GCAACGATTGGCAGCTATGG
pbp3-Rv	CGACGGTACGAACGAGTGAT
recA-Fw	TTAAACAGGCCGAATTCCAG
recA-Rv	CCGCTTTCTCAATCAGCTTC
