# Supplementary figures and images for: Spontaneous Emergence of Cefiderocol Resistance in Klebsiella pneumoniae KPC-163: Genomic and Transcriptomic Insights
Source: Antibiotics (Basel). 2025 Aug 15;14(8):832. doi: 10.3390/antibiotics14080832 (PMC12382882; doi:10.3390/antibiotics14080832)

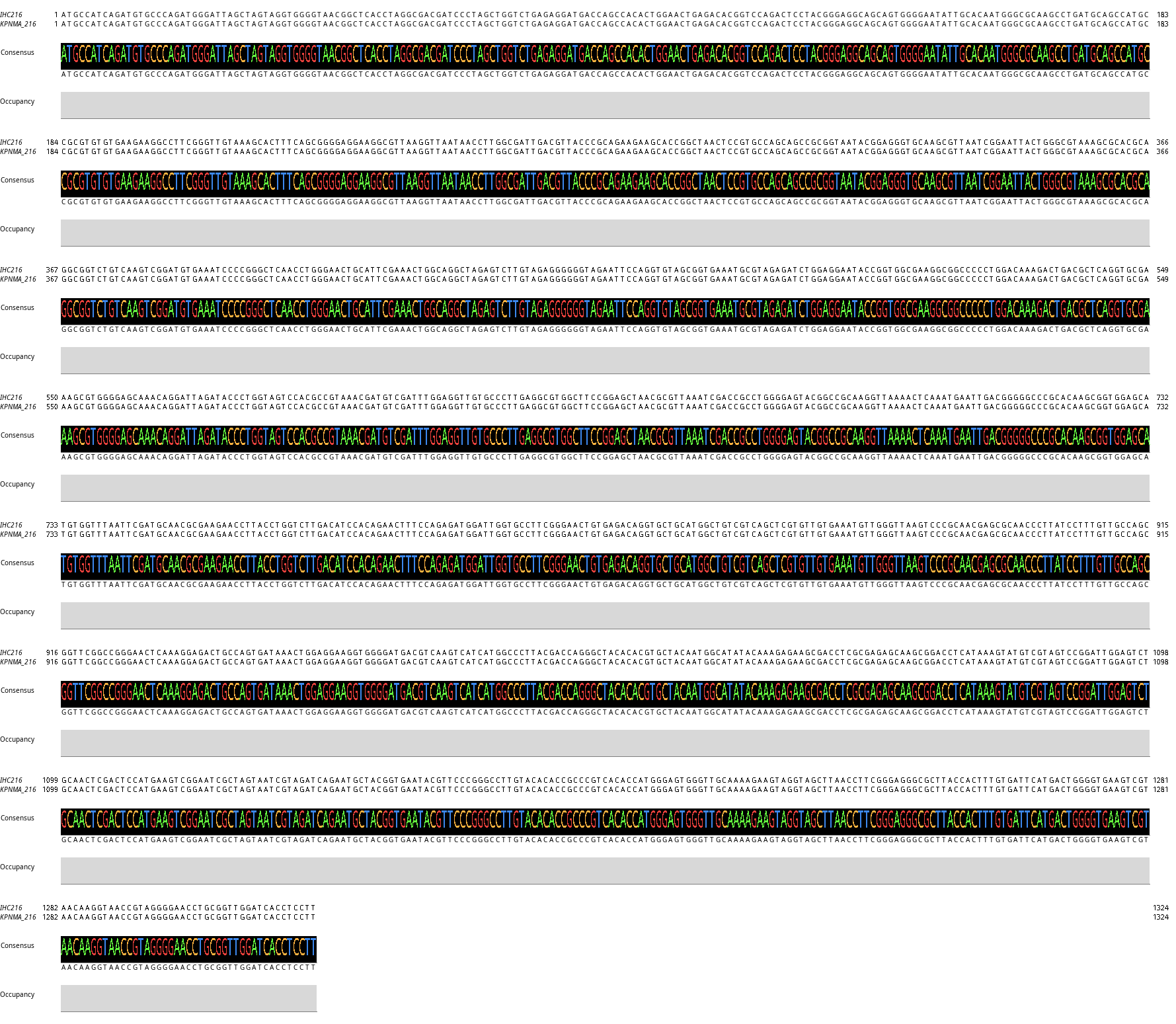

Supplement: Supplementary file 1 [file antibiotics-14-00832-s001.zip › antibiotics-3776232 FigureS4.png]

**A)****KPNMA216**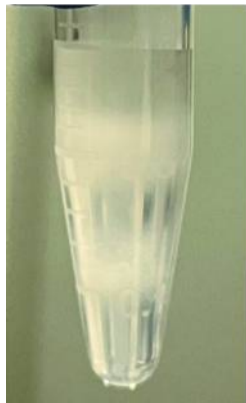**KPNMA216 IHC**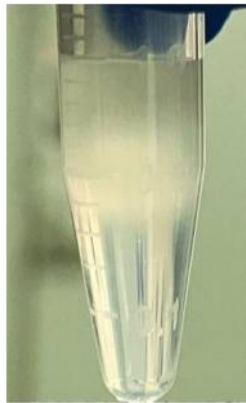**B)**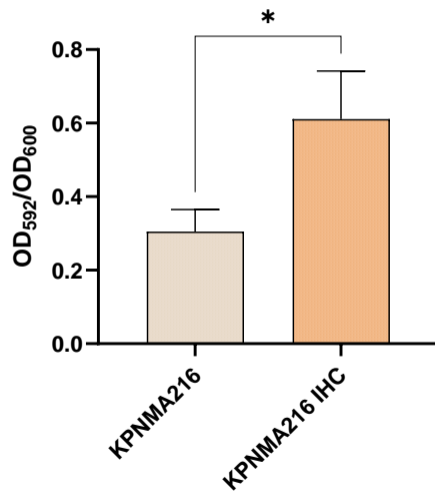**KPNMA216**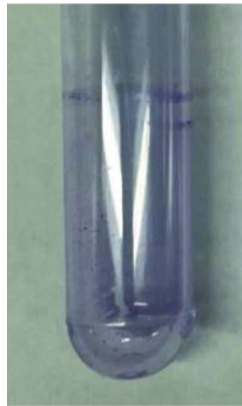**KPNMA216 IHC**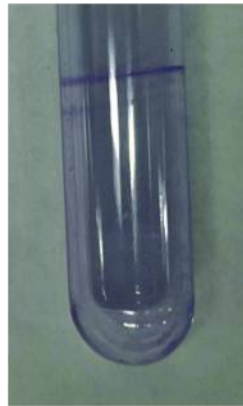

Supplement: Supplementary file 1 [file antibiotics-14-00832-s001.zip › antibiotics-3776232-figures S3.pdf]

# Sampling date

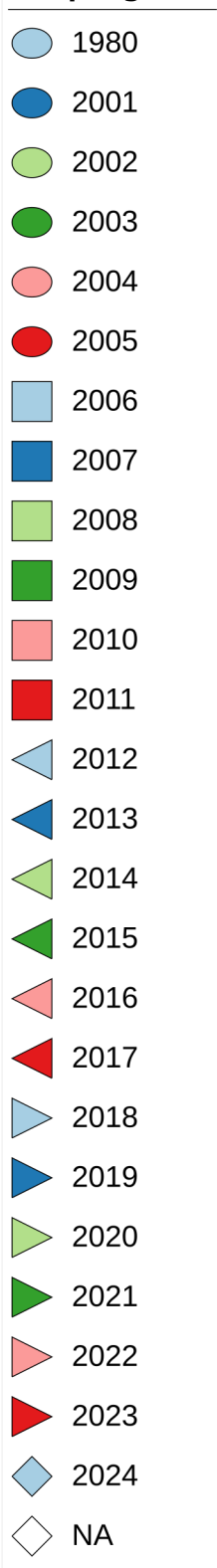

# Country

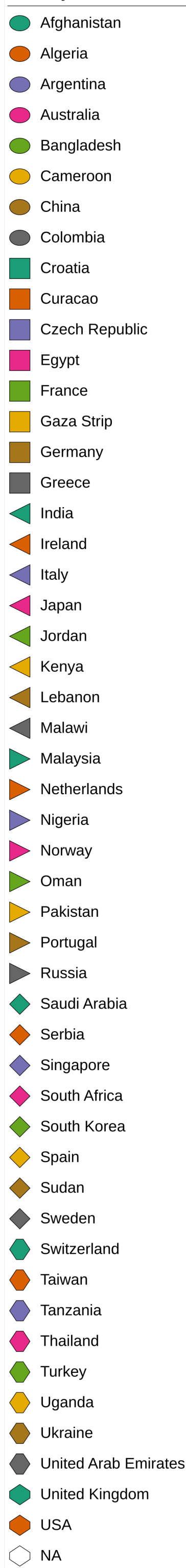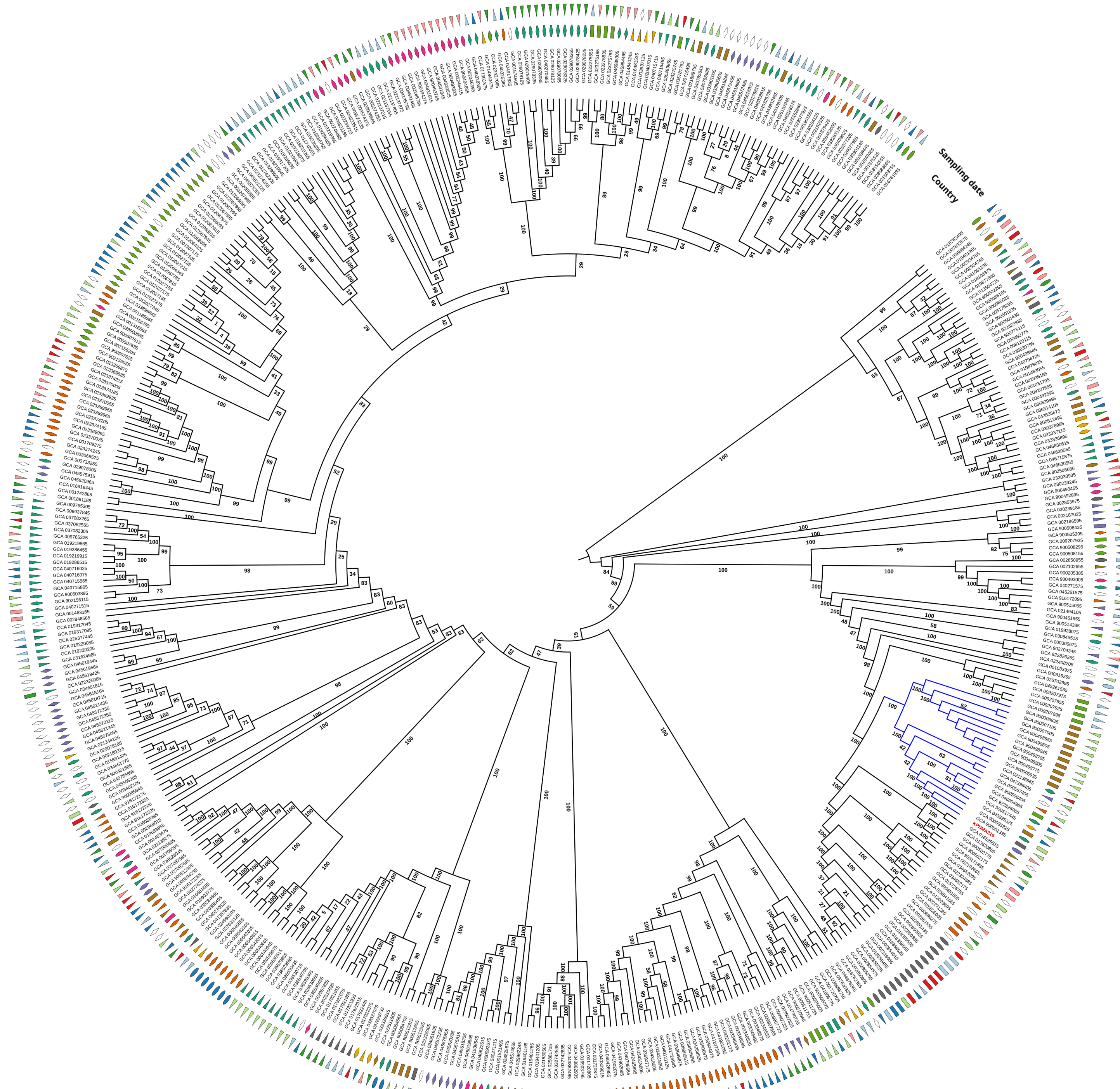

Supplement: Supplementary file 1 [file antibiotics-14-00832-s001.zip › Figure S1.pdf]

KPNMA 216  
FDC

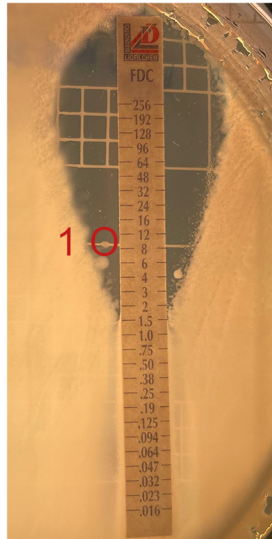

KPNMA 216  
CZA

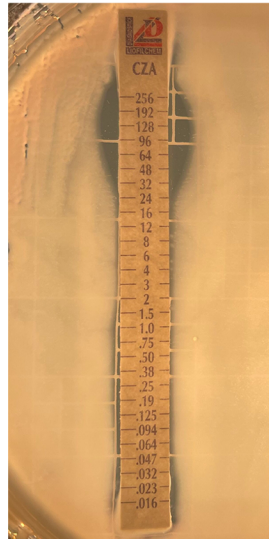

KPNMA 216  
IHC 1  
FDC

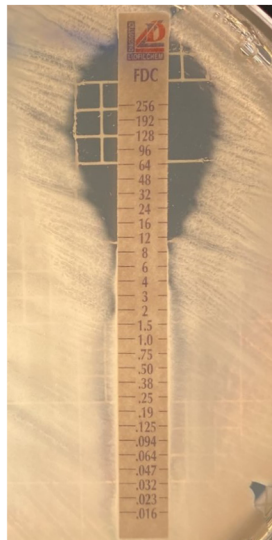

KPNMA 216  
IHC 1  
CZA

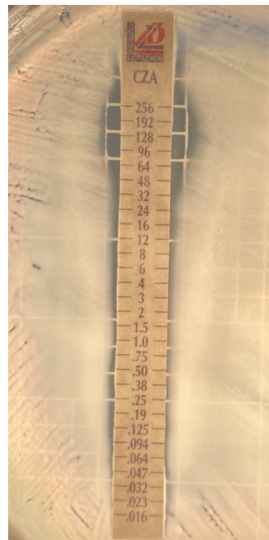

Supplement: Supplementary file 1 [file antibiotics-14-00832-s001.zip › Figure S2.png.pdf]
